# Cocrystallization of the Src-Family Kinase Hck with the ATP-Site Inhibitor A-419259 Stabilizes an Extended Activation Loop Conformation

**DOI:** 10.1021/acs.biochem.4c00323

**Published:** 2024-09-24

**Authors:** Ari M. Selzer, John J. Alvarado, Thomas E. Smithgall

**Affiliations:** Department of Microbiology and Molecular Genetics, University of Pittsburgh School of Medicine, 450 Technology Drive, Pittsburgh, Pennsylvania PA 15219, United States

## Abstract

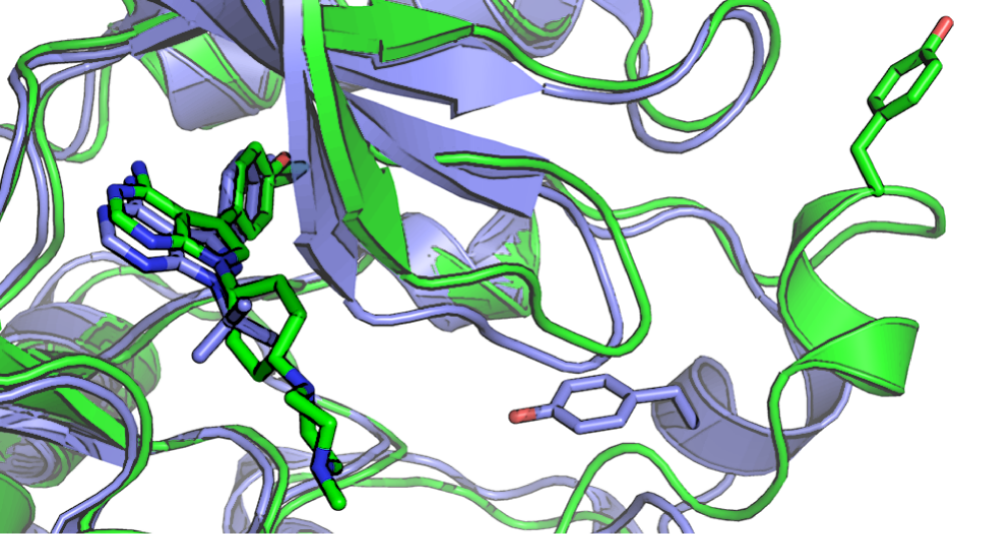

Hematopoietic cell kinase (Hck) is a member of the Src kinase family and is a promising drug target in myeloid leukemias. Here, we report the crystal structure of human Hck in complex with the pyrrolopyrimidine inhibitor A-419259, determined at a resolution of 1.8 Å. This structure reveals the complete Hck active site in the presence of A-419259, including the αC-helix, the DFG motif, and the activation loop. A-419259 binds at the ATP-site of Hck and induces an overall closed conformation of the kinase with the regulatory SH3 and SH2 domains bound intramolecularly to their respective internal ligands. A-419259 stabilizes the DFG-in/αC-helix-out conformation observed previously with Hck and the pyrazolopyrimidine inhibitor PP1 (PDB: 1QCF). However, the activation loop conformations are distinct, with PP1 inducing a folded loop structure with the tyrosine autophosphorylation site (Tyr416) pointing into the ATP binding site, while A-419259 stabilizes an extended loop conformation with Tyr416 facing out into the solvent. Autophosphorylation also induces activation loop extension and significantly reduces the Hck sensitivity to PP1 but not A-419259. In cancer cells where Hck is constitutively active, the extended autophosphorylation loop may render Hck more sensitive to inhibitors like A-419259 which prefer this kinase conformation. More generally, these results provide additional insight into targeted kinase inhibitor design and how conformational preferences of inhibitors may impact selectivity and potency.

## Introduction

The Src kinase family includes eight members in mammals (Src, Yes, Fyn, Lck, Hck, Fgr, Blk, and Lyn), which control diverse signaling pathways related to growth, differentiation, survival, and motility in almost all cell types. All family members share regulatory SH3 and SH2 domains, a catalytic kinase domain, and a negative regulatory tail.^1,2^ The SH3 and SH2 domains serve dual roles in kinase regulation and signal transduction. Previous structural studies of inhibitor-bound Src,^3^ Hck,^4^ and Fgr^5^ have shown that intramolecular contacts involving both regulatory domains stabilize a closed conformation. In this state, the SH3 domain binds to a polyproline type II (PPII) helix formed by the SH2-kinase linker, while the SH2 domain engages the tyrosine-phosphorylated negative regulatory tail. This C-terminal regulatory tail tyrosine residue is conserved across all Src-family kinases (Tyr527; residue numbering based on PDB: 2SRC(3)) and is phosphorylated by the C-terminal Src kinase (Csk).^6^ Perturbation of these regulatory contacts by intermolecular interactions with receptors, substrates, or other proteins often leads to kinase activation. In this way, kinase activity is coupled to protein–protein interactions to integrate signaling responses. Tight control of Src-family kinase activity is very important to cellular homeostasis with loss of regulation often associated with cancer cell growth, survival, and metastasis.^7^

Like many other protein kinases, the Src-family kinase domains are composed of two lobes. The smaller N-terminal lobe consists primarily of a β-sheet plus a single α-helix known as the αC-helix, which undergoes dynamic repositioning during kinase activation. The larger C-terminal lobe is exclusively helical, and the crevice between the two lobes forms the active site. Another key element of the active site is the conserved DFG motif (Asp404, Phe405, and Gly406) which, like the αC-helix, undergoes dynamic repositioning during kinase activation. Extending from the active site is another dynamic element known as the activation loop, which bears a single autophosphorylation site (Tyr416) conserved across all of the family members. When Hck adopts the autoinhibited conformation, which can be induced by orthosteric inhibitors such as PP1,^4^ Tyr416 points into the catalytic cleft and makes a polar contact with the catalytic aspartate (Asp386), which stabilizes a closed loop conformation. As part of the activation mechanism, autophosphorylation of the activation loop tyrosine disrupts this interaction and leads to an extension of the loop outward toward the solvent. In the autophosphorylated state, the DFG motif and the αC-helix rotate inward, stabilizing the active conformation. Src-family kinase autophosphorylation may occur in *trans*, with the activation loop of one kinase molecule inserting into the active site of another as observed recently in the X-ray crystal structure of an Fgr dimer.^5^ Thus, exposure of the activation loop tyrosine to solvent may be an important step in the activation mechanism.

Hck, the primary focus of this report, is expressed primarily in myeloid hematopoietic cells where it normally contributes to signal transduction associated with innate immune responses by neutrophils and macrophages.^8,9^ Because of this expression pattern, Hck dysregulation is closely associated with both chronic and acute forms of myeloid leukemia (CML and AML). Hck associates directly with Bcr-Abl, the oncogenic tyrosine kinase driver of CML, where it couples Bcr-Abl to Stat5 activation and other downstream signaling events.^10−12^ Direct phosphorylation of Bcr-Abl by Hck contributes to resistance to imatinib and other kinase inhibitors for CML.^13^ Hck is also overexpressed and constitutively active in some AML cell lines and patient samples, where high levels of expression correlate with poor survival.^14^ In both CML and AML, the orthosteric ATP-site inhibitor A-419259, also referred to as RK-20449,^15^ has been shown to suppress leukemia cell growth and survival in cell culture^11,14^ and to reverse bone marrow engraftment in mice bearing AML patient-derived xenografts.^15^ A-419259 is a pyrrolopyrimidine with nanomolar potency against Hck as well as other AML-associated Src-family members including Lyn and Fgr.^14^ The overall selectivity profile of A-419259 by KINOMEscan analysis^16^ is relatively narrow, with just 19 kinase domain interactions observed out of 468 kinases tested at the relatively high concentration of 1.0 μM.^14^ These characteristics support the further development of clinical inhibitors for AML based on the A-419259 scaffold.

A previous 3.0 Å crystal structure of Hck bound to A-419259 includes the SH3, SH2, and kinase domains as well as the negative regulatory tail (PDB: 4LUE).^17^ In this structure, the protein adopts the overall closed conformation described above. A-419259 binds at the ATP-site and stabilizes a DFG-in/αC-helix-out conformation of the kinase domain, which is incompatible with phosphotransfer. In this structure, however, electron density was not observed for the activation loop and the C-terminal part of the αC-helix, suggesting that these important regulatory elements may remain flexible, even in the crystalline state. More recently, a 1.70 Å crystal structure of the isolated Hck kinase domain with A-419259 revealed the full αC-helix and activation loop in an extended conformation (PDB: 5ZJ6).^18^ However, because the regulatory SH3 and SH2 domains may allosterically influence the conformation of the kinase domain,^19^ the determination of a high-resolution Hck structure bound to A-419259 that includes the regulatory domains is an important goal. Here, we report a new X-ray crystal structure of Hck bound to A-419259 in which the activation loop and αC-helix are fully resolved. The structure was determined at 1.8 Å resolution and revealed the autoinhibited state, with the SH3 and SH2 domains bound intramolecularly, while the kinase domain adopts the DFG-in/αC-helix-out conformation reported previously with A-419259. The αC-helix and activation loop are fully resolved, with the activation loop and Tyr416 autophosphorylation site extended into the solvent, as observed previously in the structure of the isolated kinase domain. Thus, the kinase domain can adopt a partially active conformation despite the presence of inhibitory intramolecular interactions. Interestingly, this activation loop conformation is distinct from a previous structure of Hck bound to another DFG-in/αC-helix-out inhibitor PP1 (PDB: 1QCF)^4^ in which the activation loop adopts a folded conformation and Tyr416 interacts with Asp386 as described above. Previous reports have indicated that inhibitors may bind with lower affinity when kinase domains do not adopt a preferred conformation.^19^ Thus, the unique conformation of the Hck active site induced by A-419259 in the crystal structure may more closely resemble the active conformation present in blood cancer cells, thereby explaining its remarkable potency against AML cells.^14^ In vitro kinase assays showed that A-419259 potency was less influenced by Hck autophosphorylation than that of PP1, providing support for this possibility.

## Materials and Methods

### Bacterial Expression Vector Construction

The Hck expression construct is based on the p59 form of human Hck that consists of 505 amino acids (NCBI reference sequence NP_001165604.1). For expression in *E. coli*, the nucleotide sequence encoding the SH3, SH2, linker, kinase, and C-terminal tail (Ile60 to Pro505) plus an N-terminal hexa-histidine tag was amplified by PCR and subcloned into the pET-28a(+) bacterial expression plasmid (EMD Millipore) via the Nco I and *Eco*RI restriction sites. The C-terminal tail sequence was modified to encode Tyr-Glu-Glu-Ile-Pro to ensure SH2 domain engagement as described previously.^4,20^ This vector, therefore, encodes a form of Hck in which the N-terminal His-6 tag replaces the unique domain with all other domains intact (SH3-SH2-kinase-tail). For simplicity, this form of the kinase is termed Hck throughout.

To facilitate soluble expression of downregulated Hck in *E. coli*, coexpression of human protein tyrosine phosphatase 1B (PTP1B) and the C-terminal Src kinase (Csk) was required. The coding sequence for the PTP1B catalytic domain (residues 1–283) was PCR-amplified and subcloned behind the first T7 promoter via the Nco I and *Sal*I restriction sites of pETDuet-1 (EMD Millipore). The full-length Csk coding sequence was PCR-amplified and subcloned into the pETDuet-1-PTP1B plasmid behind the second T7 promoter via the Nde I and Pac I restriction sites to yield the final pETDuet-1-PTP1B-Csk plasmid. The nucleotide sequences of the Hck, PTP1B, and Csk coding regions were confirmed by Sanger sequencing.

### Expression and Purification of Hck in *E. coli*

One Shot BL21 (DE3) STAR chemically competent *E. coli* cells (ThermoFisher) were cotransformed with the pET-28a-Hck and pETDuet-1-PTP1B-Csk vectors for the expression of Hck in its tail-phosphorylated, inactive form. Bacterial cells were grown at 37 °C with shaking in 1 L of TB with 30 μg/mL kanamycin and 50 μg/mL ampicillin. Recombinant protein expression was induced when the culture reached an OD_600_ of 0.6–0.8 by the addition of IPTG to a final concentration of 0.2 mM. Following overnight induction at 18 °C, the cells were pelleted and stored at −80 °C until lysis.

Cells were resuspended in nickel immobilized metal affinity chromatography (Ni-IMAC) binding buffer (25 mM Tris-HCl, pH 8.3, 500 mM NaCl, 20 mM imidazole, 2 mM β-mercaptoethanol (BME), and 10% glycerol) supplemented with the cOmplete EDTA-free protease inhibitor cocktail (MilliporeSigma). Cells were lysed using a microfluidizer, and the soluble protein fraction was separated via ultracentrifugation at 100 000 g for 1 h at 4 °C. The clarified lysate was loaded onto a 5 mL HisTrap HP column (Cytiva), washed, and eluted with a linear gradient of Ni-IMAC binding buffer containing 500 mM imidazole. Fractions containing Hck were identified by SDS-PAGE, pooled, and dialyzed overnight against 25 mM HEPES, pH 7.5, 1 mM EDTA, 2 mM BME, and 10% glycerol. The dialyzed sample was loaded onto a 5 mL HiTrap Blue HP affinity column (Cytiva), and Hck was eluted with dialysis buffer and 3 M NaCl via a stepwise gradient. The Hck fractions were pooled, concentrated, and dialyzed against size exclusion chromatography (SEC) buffer (20 mM Tris-HCl, pH 8.3, 150 mM NaCl, 10% glycerol, and 2 mM Tris(2-carboxyethyl) phosphine) and run over a HiLoad 16/600 Superdex 75 pg preparative SEC column (Cytiva). The Hck protein fractions were pooled, concentrated, snap-frozen in liquid nitrogen, and stored at −80 °C.

### In vitro Kinase Assays

The ADP Quest kinetic kinase assay (Eurofins) was used to measure the activity of Hck (in SEC buffer) purified from *E. coli*. The protein was diluted in ADP Quest assay buffer (15 mM HEPES, pH 7.4, 20 mM NaCl, 1 mM EGTA, 0.02% Tween-20, 10 mM MgCl_2_, and 0.1 mg/mL bovine γ-globulins) to 0.1 mg/mL, and 1000 ng were added per well in 384-well plates in a final assay volume of 20 μL. The kinase protein was then serially diluted to identify an input amount yielding approximately 50% maximum activity in the presence of an excess of ATP and substrate peptide (YIYGSFK) relative to the reported K_m_ values.^21^ This Hck input amount was identified as 125 ng/well and was used for subsequent experiments. Substrate peptide and ATP concentrations were then titrated to determine their respective K_m_ values. Hck activity was measured with and without the substrate peptide to determine total phosphorylation and autophosphorylation. Fluorescence was measured every 5 min over 3 h with a SpectraMax i3x plate reader (Molecular Devices). Fluorescence values were averaged across four technical replicates, and the autophosphorylation values were subtracted from the total phosphorylation values to obtain the peptide substrate phosphorylation rates. The linear region of each reaction progress curve was fit by regression analysis (GraphPad Prism, version 10), and the slope ± SE at each ATP and peptide substrate concentration were fit by nonlinear regression to determine the K_m_ values.

To promote autophosphorylation prior to the assessment of inhibitor potency, Hck was diluted in ADP Quest assay buffer to a concentration of 0.22 mg/mL and incubated with 100 μM ATP at room temperature for 3 h prior followed by dialysis to remove the remaining ATP. Kinase inhibitors PP1 (Cayman Chemicals) and A-419259 (Santa Cruz Biotechnology) were dissolved in ADP Quest kinase assay buffer containing 1% DMSO and diluted into the assay over a range of concentrations with the final DMSO concentration held constant at 0.1%.

### Crystallization

Tail-phosphorylated, downregulated Hck (in SEC buffer) was mixed with 10 mM A-419259 (prepared in water) and 50 mM pyrimidine diamine 1^22^ (PDA1; ChemBridge; prepared in 100% DMSO) to final concentrations of 10 mg/mL (192 μM) protein, 190 μM A-419259, 1 mM PDA1, and 2% DMSO. The protein:ligand mixture was incubated overnight at 4 °C and briefly centrifuged at 2000 g to remove particulates prior to crystallization. Crystals were grown via sitting-drop vapor diffusion at room temperature by mixing the protein solution with the mother liquor (0.2 M sodium thiocyanate, 20% (w/v) PEG 3350) in a 1:1 ratio. Crystals typically grew in about 2 weeks and were cryoprotected using 30% (v/v) ethylene glycol in the mother liquor with both ligands present followed by flash freezing in liquid nitrogen.

### X-Ray Data Collection, Processing, and Structure Determination

X-ray data were collected at the 23-ID-B beamline at the Advanced Photon Source, Argonne National Laboratory, U.S. Department of Energy. The crystal diffracted to a resolution of 1.8 Å. Data indexing, integration, and scaling were carried out using the XDS program suite.^23^ Phenix Xtriage^24^ predicted a solvent content of 0.471 and a Matthew’s coefficient (V_M_) of 2.32 Å^3^/Da, suggesting one molecule in the asymmetric unit which was consistent with the molecular replacement solution.

Phasing and structure solution were performed by molecular replacement with the program PHASER.^25^ The individual SH3, SH2, and kinase domains from a previous near-full-length Hck structure (PDB ID: 1QCF)^4^ were used as independent search models. The activation loop, C-terminal tail, and SH2-kinase linker were removed from the search model prior to phasing due to their flexible nature; these regions were built in later to fit the electron density. Iterative molecular replacement was conducted using the found solutions as fixed models in combination with search models. The molecular replacement solution was refined using rigid body, individual coordinate, individual isotropic B-factor, occupancy, simulated annealing, and TLS refinement using the program phenix.refine.^24^ Rounds of refinement and model building were conducted using the programs phenix.refine and Coot,^26^ respectively. Positive Fo-Fc electron density for the activation loop, C-terminal tail, SH2-kinase linker, and the inhibitor A-419259 were observed and used to build structural models for these features using Coot. Water molecules were added using phenix.refine, and additional water and solvent atoms were added using Coot. Final models of the X-ray structures presented in the figures were produced using PyMol (Schrödinger).

## Results and Discussion

### Expression and Purification of Hck in *E. coli*

Previous crystal structures of Hck used recombinant kinase proteins expressed in insect cell hosts.^4,27^ To adapt Hck expression to *E. coli*, we coexpressed the same form of Hck, in which an N-terminal His-tag replaces the unique region (while maintaining the SH3, SH2, and kinase domains), with the catalytic domain of the human protein-tyrosine phosphatase PTP1B and full-length human Csk. Coexpression of Hck with these two enzymes maintains a dephosphorylated activation loop (Tyr416) and a phosphorylated negative regulatory tail (Tyr527). In addition, the sequence surrounding the tail tyrosine was modified to enhance interaction with the SH2 domain (pTyr-Glu-Glu-Ile; “Hck-YEEI”) as reported previously.^20^ This approach routinely yields 3–4 mg of purified Hck per liter of bacterial culture, which was confirmed to be singly phosphorylated by mass spectrometry (calculated mass 52 083.40 Da; observed mass 52 086.07 Da).

Kinase activity was assessed using the ADP Quest kinetic kinase assay, which employs a peptide substrate and detects the generation of ADP through a series of coupled reactions that result in a fluorescent product and regeneration of ATP.^28^ Using this assay, we determined K_m_ values for both ATP (27.7 ± 18.5 μM) and the peptide substrate (28.3 ± 19.3 μM) as well as the specific activity of the kinase (32.0 ± 2.3 pmol of ADP/min/μg). These results are comparable to a previous report of Hck expressed in insect cells and assayed under similar experimental conditions (Table 1).^21^

**Table 1:** Michaelis–Menten Constants and Specific Activity of Hck Expressed in Insect Cells vs *E. coli*^a^

Kinase	Source	ATP K_m_ (μM)	Substrate K_m_ (μM)^b^	Specific activity (pmol ADP/min/μg)
Hck^c^	Insect cells	55.8 ± 18.5	83.8 ± 12.5	39.2 ± 2.8
Hck	*E. coli*	27.72 ± 18.5	28.25 ± 19.34	32.0 ± 2.3

a Values were determined using the ADP Quest kinetic kinase assay. Each value represents the mean from at least three independent determinations ± SE.

b Peptide substrate sequence: YIYGSFK.

c Values from Moroco et al.^21^.

### X-Ray Crystal Structure of Hck in Complex with A-419259

**Figure 1:**
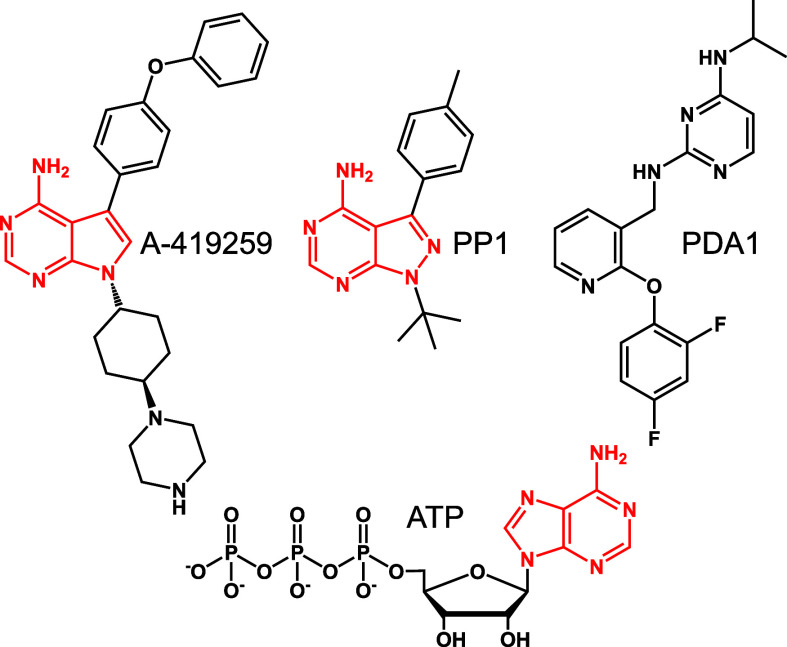
Structures of ligands referenced in this study. Shown are the structures of the orthosteric Src-family kinase inhibitors A-419259 and PP1 as well as the allosteric modulator PDA1. Highlighted in red are the pyrrolopyrimidine and pyrazolopyrimidine cores of A-419259 and PP1, respectively, which mimic the purine of ATP in the active site.

We crystallized Hck in the presence of the ATP-site inhibitor A-419259 as well as a small-molecule allosteric modulator known as pyrimidine diamine 1 (PDA1).^22^ The chemical structures of A-419259, PDA1, and PP1 are shown in Figure 1. PDA1 binds to the regulatory region of Hck^22^ and has been observed to induce global stabilization of the closed conformation in solution by hydrogen–deuterium exchange mass spectrometry (unpublished data). Therefore, PDA1 was added to the crystallization screens to promote allosteric stabilization. While strong electron density was observed for A-419259, we could not identify any density that could be attributed to PDA1. The absence of PDA1 electron density may be due to its relatively low potency for the Hck regulatory region (2-digit micromolar K_D_ value by SPR^22^), resulting in low occupancy binding within the crystal lattice.

**Figure 2:**
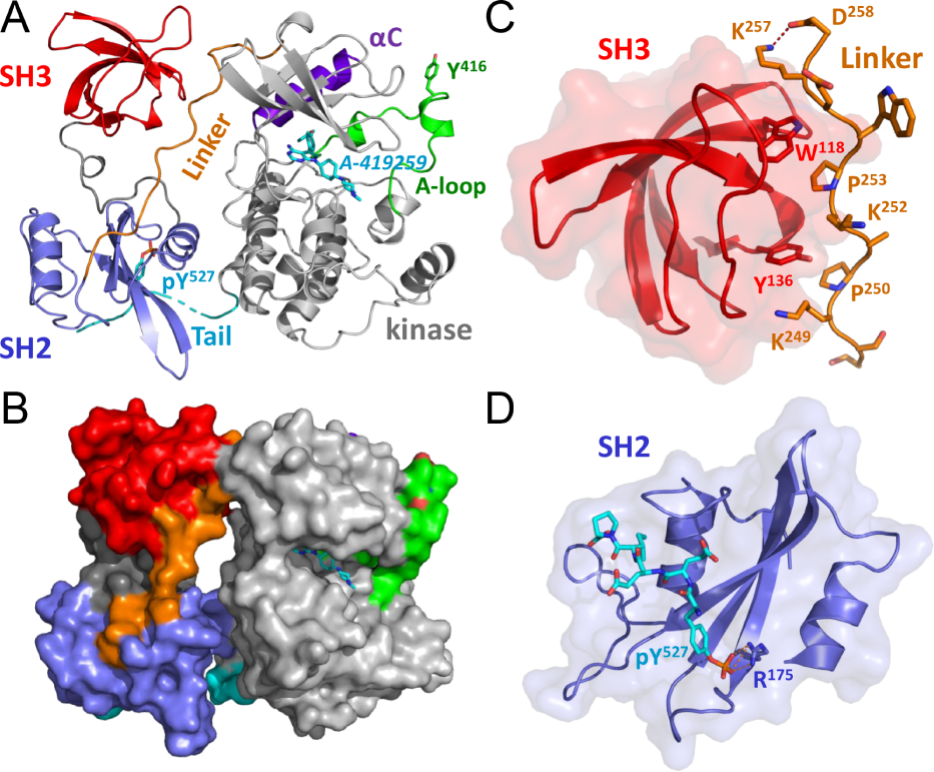
X-ray crystal structure of Hck in complex with the αC-helix-out inhibitor, A-419259. Recombinant Hck, consisting of the SH3, SH2, and kinase domains, was expressed and purified in the tail-phosphorylated (pY527), downregulated form and cocrystallized in the presence of the ATP-site inhibitor, A-419259. A) Ribbon model of the overall structure, which adopts the inactive, assembled conformation. Key structural elements include the SH3 domain (red), the SH2 domain (blue), the SH2-kinase linker (orange), the kinase domain (gray) with the N-lobe αC-helix rendered in purple, and the C-terminal tail with pTyr^527^ (cyan). The kinase domain activation loop (A-loop) and autophosphorylation site (Y416) are shown in green. The inhibitor, A-419259 (cyan), competes for ATP at the active site. B) Surface model of the structure shown in panel A, with the key features modeled in the same colors. C) Close-up view of the SH3 interface with the SH2-kinase linker. D) Close-up view of the SH2 domain bound to the C-terminal end of the negative regulatory tail, highlighting the contact between SH2 Arg175 and tail pTyr527.

The crystal structure of the Hck/A-419259 complex was determined by using molecular replacement and refined to a final resolution of 1.8 Å. Details of X-ray data collection and structure refinement statistics are presented in Table 2. Electron density was observed for residues His81-Pro531. The kinase crystallized in the closed conformation with the SH3 domain interacting with the SH2-kinase linker and the SH2 domain bound to the C-terminal tail phosphotyrosine (Figures 2A and 2B). In the kinase domain, the complete αC-helix and activation loop are clearly visible with the autophosphorylation site (Tyr416) extending outward from a short α-helix. The SH3 domain engages the linker through typical hydrophobic grooves on the binding surface. SH3 residues Trp118 and Tyr136 make contacts with linker Pro253 and Pro250 that stabilize a PPII helix that forms the internal SH3 ligand (Figure 2C). The SH2 domain engages the tail phosphotyrosine with the positively charged side chain of Arg175 in the phosphotyrosine binding pocket of the SH2 domain forming a salt bridge with the pTyr527 phosphate group in the C-terminal tail (Figure 2D). Previous studies have shown that mutagenesis of tail Tyr527, SH3 Trp118, or linker prolines all cause Hck activation in cells, demonstrating the importance of these intramolecular interactions in the regulation of kinase activity.^29,30^

**Table 2:** X-Ray Data Collection and Refinement Statistics for the Hck: A-419259 Crystal Complex^a^

Hck with A-419259	
Wavelength (Å)	1.03
Resolution range	41.08–1.8 (1.864–1.8)
Space group	P 21 21 21
Unit cell	43.346 85.025 128.798 90 90 90
Total reflections	320408 (30593)
Unique reflections	44980 (4385)
Multiplicity	7.1 (7.0)
Completeness (%)	99.89 (99.23)
Mean I/sigma(I)	9.88 (0.62)
Wilson B-factor	33.27
R-merge	0.148 (2.891)
R-meas	0.1596 (3.128)
R-pim	0.05885 (1.178)
CC1/2	0.998 (0.241)
CC*	0.999 (0.623)
Reflections used in refinement	44949 (4357)
Reflections used for R-free	1997 (192)
R-work	0.1994 (0.3717)
R-free	0.2193 (0.3796)
CC(work)	0.964 (0.548)
CC(free)	0.971 (0.653)
Number of non-hydrogen atoms	3944
macromolecules	3612
ligands	113
Solvent	219
Protein residues	450
RMS (bonds)	0.013
RMS (angles)	1.58
Ramachandran favored (%)	97.08
Ramachandran allowed (%)	2.92
Ramachandran outliers (%)	0
Rotamer outliers (%)	4.2
Clashscore	5.89
Average B-factor	45.67
macromolecules	45.55
Ligands	49.45
Solvent	45.69
Number of TLS groups	7

a Statistics for the highest-resolution shell are shown in parentheses.

**Figure 3:**
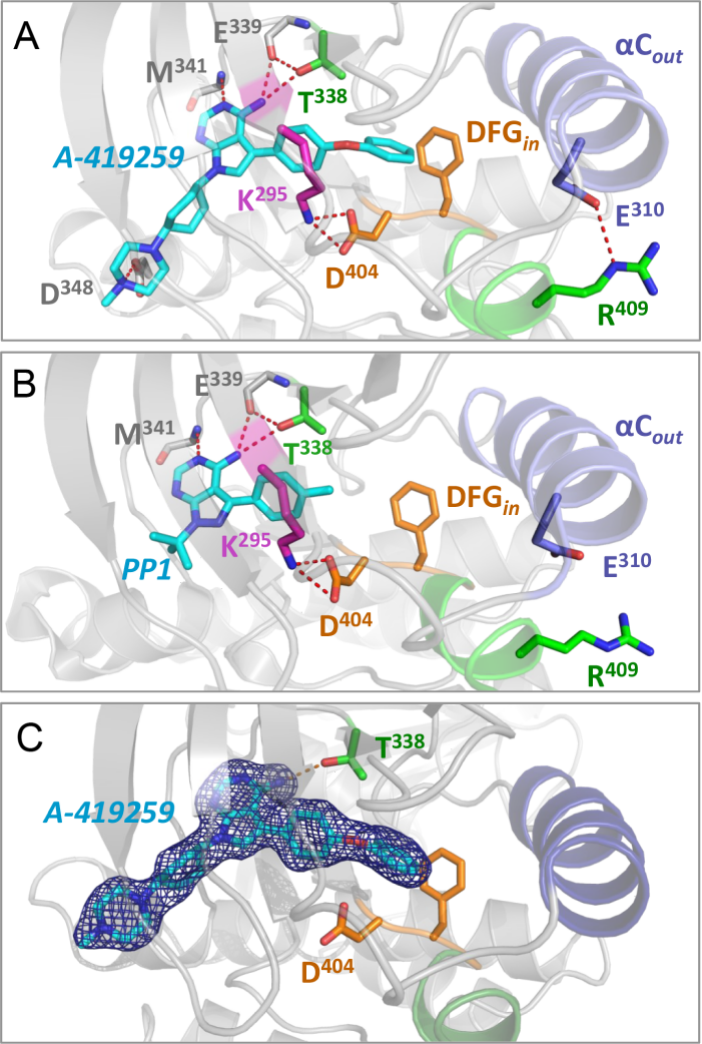
Comparison of A-419259 and PP1 binding sites. A) Close-up view of the A-419259 binding site in the Hck kinase domain. A-419259 binding induces outward rotation of the αC-helix such that Glu310 cannot ion-pair with Lys295, an interaction essential for kinase activity. Instead, Glu310 makes polar contact with Arg409 from the activation loop. The Glu-Phe-Gly motif is rotated inward (DFG*_in_*), with Asp404 contacting Lys295. The aminopyrimidine of the inhibitor makes a polar contact with the gatekeeper residue (Thr338) as well as the side chains of the hinge residues Glu339 and Met341, while the methylpiperazine makes a contact with Asp348. B) The pyrazolopyrimidine inhibitor PP1 also induces an αC-helix-out/DFG-in conformation of the Hck active site, making similar contacts with the hinge and the gatekeeper residues. In this case, Glu310 is more than 5 Å distant from Arg409, preventing hydrogen bond formation. C) Well-ordered 2F_O_-F_C_ electron density (blue mesh, contoured at 1σ) for A-419259 bound in the Hck active site.

A prior structure of Hck bound to A-419259 (PDB: 4LUE)^17^ was determined at 3.0 Å and adopts the closed conformation but lacks electron density for the activation loop or distal end of the αC-helix, as described above. An earlier structure of the same form of recombinant Hck bound to the pyrazolopyrimidine inhibitor PP1 also adopts the closed conformation (PDB: 1QCF).^4^ Superposition of our Hck structure with those of 4LUE and 1QCF indicates very similar overall conformations, as reflected in overall RMSD values of 0.766 Å vs chain A of 4LUE and 1.205 Å vs 1QCF. Both A-419259 (Figure 3A) and PP1 (Figure 3B) induce the DFG-in/αC-helix-out conformation of the active site, with Asp404 in the DFG motif forming a salt bridge with Lys295. Both inhibitors also form hydrogen bonds with the gatekeeper residue, Thr338, as well as the main chain of Met341, a nearby hinge residue. While both inhibitors occupy nearly identical positions within the active site, A-419259 has a much more extended structure than PP1, with a cyclohexylmethylpiperazine group that contacts Asp348 and a methylphenoxybenzene moiety that extends further into the hydrophobic pocket. A 2F_0_-F_c_ map shows an exceptionally good fit for A-419259 in the binding pocket (Figure 3C).

### A-419259 Stabilizes a Unique Conformation of the Hck Activation Loop

**Figure 4:**
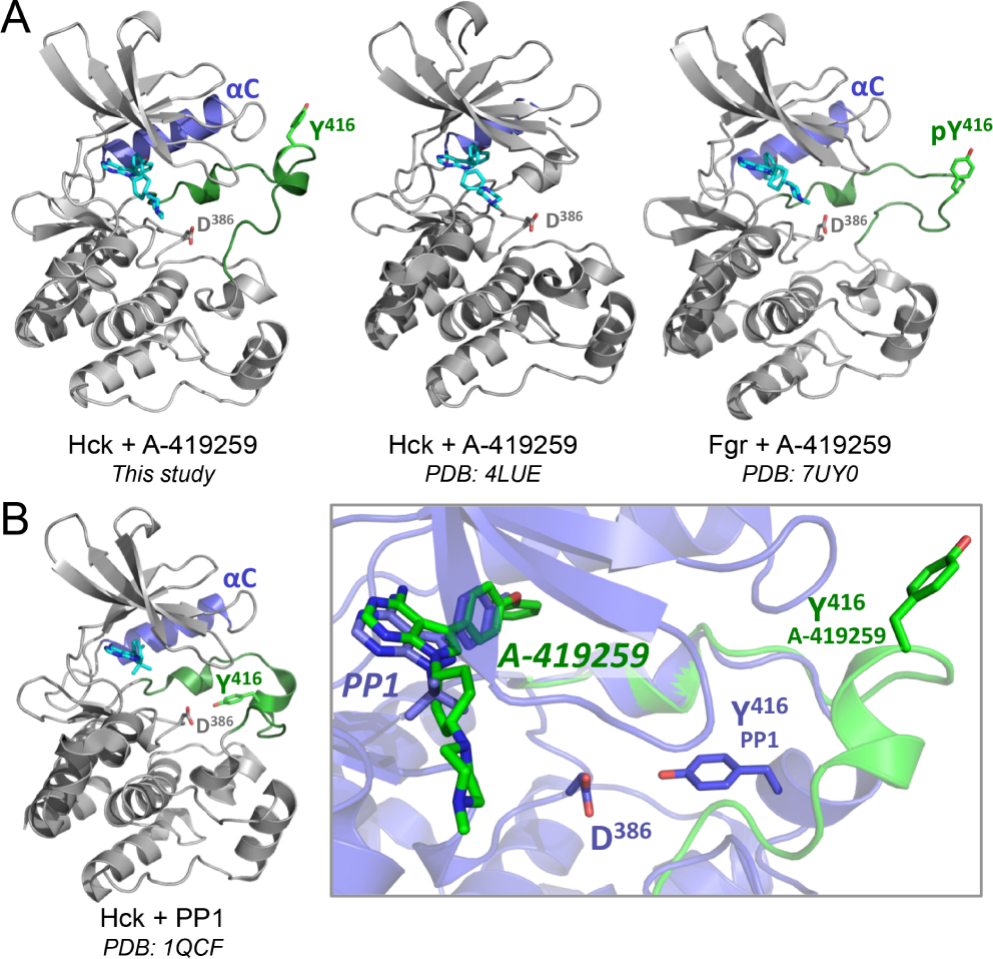
Stabilization of the Hck kinase domain αC-helix and activation loop by A-419259 vs PP1. A) A-419259-bound kinase domain structures of Hck (this study and PDB: 4LUE) and Fgr (PDB: 7UY0). In this study, the activation loop (green) and autophosphorylation site (Tyr416) adopt an extended conformation, with the complete αC-helix rotated outward. Both features are missing in the previous Hck structure. In Fgr, the activation loop is also extended but lacks a secondary structure. B) Left: Hck with PP1 bound to the active site (PDB: 1QCF). Right: Alignment of the Hck activation loop conformations induced by PP1 (blue ribbon) and A-419259 (green ribbon). Both activation loops adopt a partial helical structure. The activation loop from the A-419259 structure extends outward, with the autophosphorylation site (Tyr416) pointing away from the active site. In contrast, the loop induced by PP1 is folded inward with Tyr416 approaching the catalytic aspartate (Asp386).

One distinctive feature of our A-419259-bound Hck structure is the position of the activation loop, which includes residues Asp404-Pro425. A-419259 induces an extended activation loop conformation with the tyrosine autophosphorylation site facing away from the active site and into the solvent (Figure 4A). This activation loop conformation was also seen with a structure of the isolated Hck kinase domain in the presence of A-419259 (PDB: 5ZJ6),^18^ indicating that the extended activation loop conformation does not require the regulatory domains. Alignment of the 5ZJ6 kinase domain-only structure with the kinase domain from our structure revealed an RMSD of less than 0.5 Å, demonstrating that the two kinase domains adopt virtually identical conformations. Specifically, there are two chains in the 5ZJ6 structure, and their respective RMSD values compared to those of the kinase domain in our structure are 0.452 and 0.449. The conformations of the activation loops (amino acids 404–425) are similarly conserved, with RMSD values of 0.305 and 0.299, respectively. A similar activation loop conformation is observed in the crystal structure of the related Src-family kinase Fgr when bound to A-419259 (PDB: 7UY0), suggesting that this effect of the inhibitor is not unique to Hck (Figure 4A).^5^ While A-419259 induces an extended loop conformation in both Hck and Fgr, the conformation of each loop is distinct (Figure 4A), giving credence to the potential flexibility of the loop in the extended state. In addition, the amino acid sequence of the Fgr activation loop is different from Hck and all other members of the Src kinase family, with a proline residue at the +2 position relative to the tyrosine autophosphorylation site.^31^ The presence of this proline may prevent the formation of the α-helix observed with Hck.

Although PP1 and A-419259 induce the same conformational changes to the Hck active site (DFG-in/αC-helix-out), PP1 induces a distinct conformation of the activation loop. In this case, the activation loop tyrosine (Tyr416) tucks into the active site, where it is positioned to make a hydrogen bond with the catalytic aspartate (Asp386; Figure 4B). This folded activation loop structure along with the Tyr416–Asp386 interaction is associated with the inactive state of the kinase domain, while the extended conformation seen with A-419259 implies the potential for intermediate conformations. Understanding the complex activation pathways of kinase domains as dynamic instead of simple on–off switches may open new avenues for therapeutic development.^32^

### A-419259 Inhibition Is Less Sensitive to Kinase Activation than PP1

While the purine-like cores of both PP1 and A-419259 (Figure 1) occupy the same kinase domain binding site and induce the DFG-in/αC-helix-out conformation, their effects on the activation loop are dramatically different in the crystalline state (Figure 4B). The extended activation loop conformation induced by A-419259 may represent an intermediate between active and inactive conformations of the kinase domain, where autophosphorylation is also known to cause the extension of this loop as exemplified by the structure of the autophosphorylated kinase domain of Lck (PDB: 3LCK).^33^ This observation suggested that Hck autophosphorylation may have a lesser impact on the inhibition of Hck by A-419259 compared with PP1, where inhibitor binding causes the folding of the activation loop into the active site.

**Figure 5:**
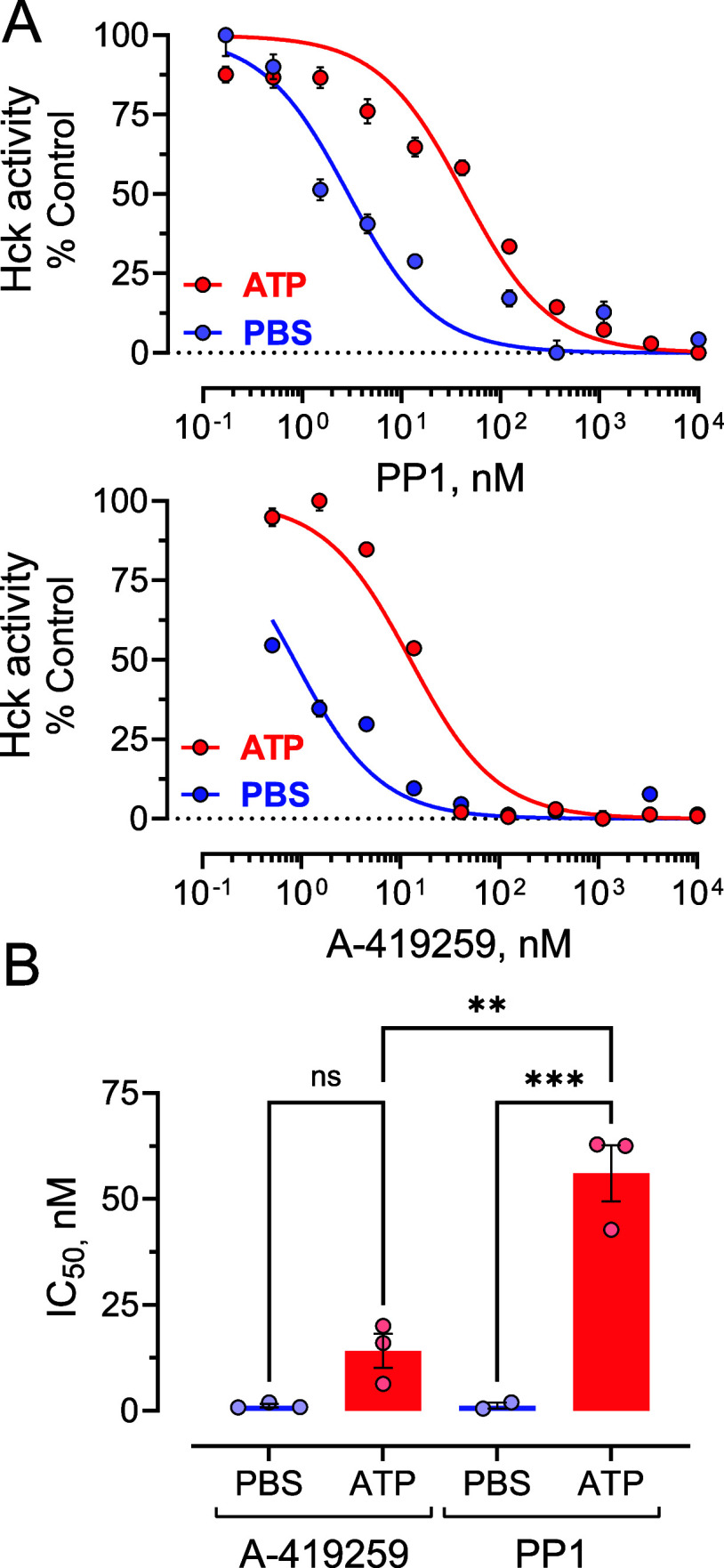
Effect of autophosphorylation on Hck sensitivity to ATP-site inhibitors. These experiments used 3-domain Hck (SH3-SH2-kinase) that was initially purified in the tail-phosphorylated, downregulated form, the same preparation used for X-ray crystallography. Hck was preincubated with ATP/Mg++ to induce autophosphorylation of the activation loop or with PBS as a control. Each preparation was then assessed for activity using the ADP Quest kinetic kinase assay in the presence of the concentrations of either PP1 or A-419259 shown. Data were normalized to the steady-state reaction rates observed in the absence of inhibitor and best-fit by nonlinear-regression analysis to obtain IC_50_ values. A) Representative concentration–response curves. Each data point represents the average of four technical replicates ± SE. B) IC_50_ values from replicate assays. Bar heights indicate the mean value ± SE. Statistical significance was evaluated by two-way ANOVA; ***, *p* < 0.001; **, *p* < 0.01; ns, not significant.

To explore this idea, we preincubated Hck with ATP/Mg^2+^ to induce autophosphorylation or with an equal volume of PBS as a negative control. Each kinase preparation was then assayed over a range of PP1 and A-419259 concentrations to assess the inhibitor potency. In the absence of the inhibitors, preincubation with ATP resulted in a shorter lag time to steady-state reaction velocity, consistent with autophosphorylation as observed previously.^21^ Concentration–response assays (Figure 5A) using Hck that was preincubated with PBS yielded similar IC_50_ values for both inhibitors (1.23 ± 0.39 nM for A-419259 and 1.22 ± 0.72 nM for PP1; mean ± SE). Prior autophosphorylation of Hck reduced the apparent potency of both inhibitors, yielding IC_50_ values of 14.12 ± 7.02 nM for A-419259 and 56.05 ± 6.64 nM for PP1. Replicate determinations of the IC_50_ values enabled a statistical comparison of the results for each inhibitor (Figure 5B). The 46-fold shift in potency observed with autophosphorylated Hck and PP1 was statistically significant (*p* < 0.001) while the 12-fold difference for A-419259 was not. In addition, the decrease in sensitivity of autophosphorylated Hck to PP1 was greater than that observed with A-419259 (*p* < 0.01). The negative impact of autophosphorylation on PP1 potency may reflect structural changes that impair the activation loop from adopting the compact conformation observed in the PP1 cocrystal structure as well as the inward rotation of the αC-helix. A-419259, on the other hand, prefers the extended conformation of the activation loop, potentially leaving only the energetic barrier of the inward-facing αC-helix to bind at the active site.

### Summary and Conclusions

The lack of electron density for the activation loop and complete αC-helix in a previous crystal structure of Hck bound to A-419259 (PDB: 4LUE)^17^ suggested that this potent inhibitor alone may not stabilize a single conformation of these dynamic active site elements. Here, we report the cocrystallization of Hck in the presence of A-419259 and the allosteric modulator PDA1,^22^ which may have helped to stabilize Hck during crystallization. Hck is present in a single-crystalline state in which the αC-helix and activation loop are both clearly resolved. In the presence of A-419259, the activation loop of Hck is extended into the solvent with the autophosphorylation site, Tyr416, facing away from the active site. This conformation is in marked contrast to the structure of Hck with PP1, where the activation loop is tucked into the ATP-site and stabilized by the Tyr416–Asp386 interaction. We propose that this preferred A-419259 conformation represents a unique intermediate between active and inactive conformations of Hck and possibly other Src-family kinases, while PP1 induces a more fully downregulated kinase domain structure. This possibility is supported by the impact of Hck autophosphorylation on inhibitor potency with a significant impact on PP1 but less so on A-419259. Hck and other Src-family members are often overexpressed and constitutively activated in multiple forms of cancer. A-419259 and related orthosteric inhibitors that induce an extended activation loop conformation as part of their preferred binding mode may be more effective in cancer cells, where Src-family kinases are autophosphorylated with the loop in the extended state.

## Notes

The final crystal coordinates for the structure of Hck (UniProt ID: **P08631**) bound to A-419259 have been deposited in the RCSB Protein Data Bank with accession code **9BYJ** and will be released upon publication.
